# Treatment of patients with *BRAF*^*V600E*^-mutated metastatic colorectal cancer after progression to encorafenib and cetuximab: data from a real-world nationwide dataset

**DOI:** 10.1016/j.esmoop.2024.102996

**Published:** 2024-04-12

**Authors:** M.M. Germani, G. Vetere, F. Santamaria, R. Intini, F. Ghelardi, M. Bensi, A. Boccaccino, A. Minelli, M. Carullo, P. Ciracì, A. Passardi, S. Santucci, R. Giampieri, M. Persano, E. Fenocchio, A. Puccini, S. Lonardi, F. Pietrantonio, L. Salvatore, C. Cremolini

**Affiliations:** 1 Department of Translational Research and New Technologies in Medicine, University of Pisa, Pisa; 2 Unit of Medical Oncology 2, Azienda Ospedaliero-Universitaria Pisana, Pisa; 3 Department of Experimental Medicine, Sapienza University of Rome, Rome; 4 Department of Oncology, Veneto Institute of Oncology IOV - IRCCS, Padua; 5 Department of Medical Oncology, Fondazione IRCCS Istituto Nazionale dei Tumori, Milan; 6 Medical Oncology Unit, Comprehensive Cancer Center, Fondazione Policlinico Universitario Agostino Gemelli IRCCS, Rome; 7 Medical Oncology Unit, Università Cattolica del Sacro Cuore, Rome; 8 Oncology Unit, Ravenna Hospital, AUSL Romagna, Ravenna; 9 Department of Medical Oncology, University Campus Bio-Medico, Rome; 10 Department of Medical Oncology, IRCCS Istituto Romagnolo per lo Studio dei Tumori (IRST) “Dino Amadori”, Meldola; 11 Oncologia Clinica, Dipartimento Scienze Cliniche e Molecolari, Università Politecnica delle Marche, Torrette di Ancona, Ancona; 12 Oncologia Clinica, Azienda Ospedaliero-Universitaria delle Marche, Ancona; 13 Medical Oncology, University Hospital of Cagliari, Cagliari; 14 Medical Oncology, University of Cagliari, Cagliari; 15 Department of Medical Oncology, University of Turin Medical School, Candiolo Cancer Institute, FPO, IRCCS, Candiolo, Turin; 16 Medical Oncology and Hematology Unit, IRCCS Humanitas Research Hospital, Humanitas Cancer Center, Rozzano, Milan, Italy

**Keywords:** *BRAF*^*V600E*^ mutation, metastatic colorectal cancer, real-world analysis

## Abstract

**Background:**

Targeted therapy (TT) with encorafenib and cetuximab is the current standard for patients with *BRAF*^*V600E*^*-*mutated metastatic colorectal cancer (mCRC) who received one or more prior systemic treatments. However, the median progression-free survival (mPFS) is ∼4 months, and little is known about the possibility of administering subsequent therapies, their efficacy, and clinicopathological determinants of outcome.

**Methods:**

A real-world dataset including patients with *BRAF*^*V600E*^*-*mutated mCRC treated with TT at 21 Italian centers was retrospectively interrogated. We assessed treatments after progression, attrition rates, and outcomes.

**Results:**

Of the 179 patients included, 85 (47%), 32 (18%), and 7 (4%) received one, two, or three lines of treatment after TT, respectively. Those receiving TT in the second line were more likely to receive at least one subsequent therapy (53%), as compared with those treated with TT in the third line or beyond (30%; *P* < 0.0001), and achieved longer postprogression survival (PPS), also in a multivariate model (*P* = 0.0001). Among 62 patients with proficient mismatch repair/microsatellite stable (pMMR/MSS) tumors receiving one or more lines of treatment after second-line TT, combinatory chemotherapy ± anti-vascular endothelial growth factor (anti-VEGF) was associated with longer PFS and PPS as compared with trifluridine–tipiracil or regorafenib (mPFS: 2.6 versus 2.0 months, *P* = 0.07; PPS: 6.5 versus 4.4 months, *P* = 0.04).

**Conclusions:**

Our real-world data suggest that TT should be initiated as soon as possible after the failure of first-line treatment in *BRAF*^*V600E*^-mutated mCRC. Among patients with pMMR/MSS tumors, combinatory chemotherapy ± anti-VEGF appears the preferred treatment choice after TT failure.

## Highlights

• Little evidence is available regarding treatments and determinants of outcome after progressive disease (PD) to encorafenib + cetuximab in patients with *BRAF*^*V600E*^*-*mutated mCRC.
• About 47% and 18% of patients received one or more and two or more lines of therapy after encorafenib + cetuximab, respectively.
• Receiving encorafenib + cetuximab after the second line and the presence of ascites at PD were independently associated with shorter survival.
• After PD, combinatory chemotherapy ± anti-VEGF was the most efficacious treatment regimen.

## Introduction

*BRAF*^*V600E*^ mutation is detected in ∼4%-9% of metastatic colorectal cancers (mCRCs) and defines a molecular subgroup with high biological aggressiveness, chemo-refractoriness, and dismal prognosis.^1^, ^2^, ^3^ With the exception of patients with deficient mismatch repair/microsatellite instable-high (dMMR/MSI-H) tumors (≈30% of *BRAF*^*V600E*^*-*mutated patients), who may achieve long-term benefit from immunotherapy,^4^^,^^5^ the upfront treatment of this molecular subgroup consists of combinatory cytotoxic regimens with fluoropyrimidines, oxaliplatin, and/or irinotecan, plus the anti-vascular endothelial growth factor (anti-VEGF) monoclonal antibody bevacizumab, when feasible.^6^^,^^7^

After disease progression, at least one-third of patients do not receive any subsequent systemic treatment, mainly because of rapid clinical deterioration.^8^ For the others, the targeted therapy (TT) consisting of the BRAF inhibitor encorafenib plus the anti-epidermal growth factor receptor (anti-EGFR) cetuximab is the current standard of care, based on the results of the randomized phase III BEACON CRC trial that demonstrated a statistically and clinically significant benefit from encorafenib plus cetuximab over standard irinotecan-based chemotherapy plus cetuximab in terms of overall survival (OS), overall response rate, and progression-free survival (PFS) in pretreated patients with *BRAF*^*V600E*^*-*mutated mCRC.^9^^,^^10^ The triple-targeted strategy incorporating the MEK inhibitor binimetinib yielded similar efficacy but a less favorable toxic profile; therefore the doublet strategy is currently recommended.^9^^,^^10^ Nonetheless, the vast majority of patients will eventually experience disease progression (PD), and little is known about potential treatment strategies after the failure of TT, including their feasibility and efficacy. Indeed, current therapeutic recommendations are mainly extrapolated from clinical trials conducted in molecularly unselected populations and where *BRAF*^*V600E*^*-*mutant patients were therefore largely underrepresented.^11^, ^12^, ^13^

Drawing from these considerations, we collected data about therapies received after progression to TT in a large Italian multicentric cohort of patients with *BRAF*^*V600E*^*-*mutated mCRC, with the aim of exploring the current treatment choices, their sequence and efficacy, as well as clinical factors affecting prognosis after TT failure.

## Methods

### Study population

This is a retrospective cohort study focused on patients with *BRAF*^*V600E*^*-*mutant mCRC who experienced PD during or after encorafenib plus cetuximab ± binimetinib, between May 2019 and April 2023. All patients received TT in a real-world setting at 21 Italian institutions. Binimetinib was initially recommended as part of the TT until January 2020 after which only the doublet encorafenib plus cetuximab was recommended. The study was approved by the ethical review board of the coordinating center (ID: 3920/2013) and was conducted in accordance with the ethical principles for medical research involving human participants adopted in the Declaration of Helsinki.

### Reporting of data about treatments after TT

The number of patients receiving at least one cycle of any treatment after progression to TT was calculated and the attrition rate per each line was defined as the ratio between the number of patients treated in each line to the number of patients treated in the previous one. Per each line, therapies were grouped as follows: oxaliplatin based, irinotecan based, regorafenib, trifluridine–tipiracil, and immunotherapy. Other treatments were classified as ‘other’. Data visualization was carried out with the R package (R Foundation, Vienna, Austria) ‘plotly’ (Plotly Technologies Inc., Montréal, QC; https://plot.ly).

### Efficacy outcomes and statistical analysis

The median follow-up was calculated from the date of PD during or after TT using the reverse Kaplan–Meier method. Postprogression survival (PPS) was defined as the time interval between the date of PD to TT and death. Patients still alive at the time of the analysis were censored at the last date when they were known to be alive. PFS per each line was defined as the time from the beginning of that line of treatment to the evidence of PD or death, whichever occurred first. Patients who did not experience PD and were still alive at the date of analysis were censored at the last date when they were known to be alive. The overall response rate per each line was defined as the ratio between the number of patients achieving partial or complete response according to RECIST criteria version 1.1 to the overall number of treated patients.

PFS and PPS were plotted using the Kaplan–Meier estimates method and survival curves were compared with the log-rank test. The hazard ratio (HR) and 95% confidence interval (CI) were estimated with a Cox proportional hazard model. The McNemar test was used to compare the attrition rates between patients receiving TT in the second and third lines and beyond, respectively. The impact of clinical, molecular, and pathological features on PPS was assessed. A Cox proportional hazard model was developed to investigate independent predictors of PPS. Covariates associated with PPS with *P* < 0.10 at univariate analyses were used to build a multivariable Cox proportional hazard model. Statistical significance was set at a *P* value of 0.05. All analyses were carried out with MedCalc statistical software version 22.002 (MedCalc Software Ltd, Ostend, Belgium; https://www.medcalc.org; 2023) and RStudio version 4.1.1 [Posit team (2023). RStudio: Integrated Development Environment for R; Posit Software, PBC, Boston, MA; http://www.posit.co/].

## Results

A total of 179 patients were eligible. Their demographic and clinical characteristics and MSI/MMR status are summarized in Supplementary Table S1, available at https://doi.org/10.1016/j.esmoop.2024.102996. The median age at the time of CRC diagnosis was 64 years and 106 patients (59%) were females;119 (66%) primary tumors were right-sided and metastases were synchronous in 128 (71%) cases. Mucinous histology was detected in 61 (36%) tumors. Nineteen (11%) tumors were dMMR/MSI-H. Previous treatments included oxaliplatin and irinotecan in 165 (92%) and 107 (60%) patients, respectively, while 136 (76%) and 12 (7%) patients had been previously exposed to bevacizumab or anti-EGFR agents, respectively. Trifluridine–tipiracil and regorafenib were administered in 9 (5%) and 5 (3%) patients, respectively. Five patients with dMMR/MSI-H tumors (3%) received immune checkpoint inhibitors (ICIs) before TT. With regard to first-line regimens, triplets, doublets, monochemotherapy, and immunotherapy were administered to 57 (32%), 109 (61%), 12 (7%), and 1 (<1%) patients, respectively.

A total of 133 (75%) patients received encorafenib plus cetuximab in the second line, and the remaining 46 (25%) in the third or subsequent line. A targeted triplet with an MEK inhibitor was administered in 38 (21%) patients. As many as 114 (64%) patients achieved disease control during TT, including 34 (19%) objective responses. Primary resistance was observed in 64 (36%) patients. The median PFS (mPFS) with TT was 4.7 months (95% CI 4.1-5.2 months). At the time of PD to TT, peritoneal metastases and ascites were detected in 109 (68%) and 54 (35%) patients, respectively, with metastases involving three or more organs in 103 (64%) cases, including sites not affected at the beginning of TT in 57 (36%) patients.

**Figure 1:**
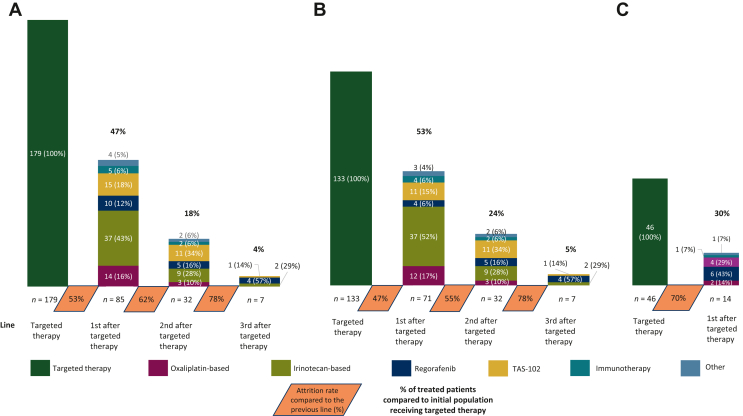
**Funnel plot of treatments received after progression to TT in (A) the overall population, (B) in those receiving TT in the second line, and (C) in the third line or beyond**. TAS-102, trifluridine–tipiracil; TT, targeted therapy.

At the time of data cut-off (26 October 2023), 85 (47%), 32 (18%), and seven (4%) patients had received at least one, two, or three lines of systemic treatment after TT, corresponding to attrition rates across the first, second, and third lines after TT of 53%, 62%, and 88%, respectively (Figure 1A). Irinotecan- and oxaliplatin-based treatments were the preferred options after TT in 37 (43%) and 14 (16%) patients, respectively, followed by trifluridine–tipiracil, regorafenib, immunotherapy, and other treatments in 15 (15%), 10 (12%), 5 (6%), and 4 (5%) patients, respectively. Trifluridine–tipiracil and regorafenib were the treatments of choice in the second (50%) and third (86%) line after progression to TT. Overall, eight patients (5%) with dMMR/MSI-H tumors naive to ICIs received immunotherapy after TT.

Remarkably, 71 (53%) out of 133 patients receiving TT in the second line were treated with at least one systemic treatment after PD, with irinotecan- and oxaliplatin-based regimens administered to 49 (69%) patients, followed by regorafenib or trifluridine–tipiracil (21%) (Figure 1B). By contrast, only 14 (30%) out of 46 patients receiving TT after the second line were treated with at least one line of systemic treatment after PD (*P* < 0.0001), including 10 patients (71%) receiving trifluridine–tipiracil or regorafenib and two patients treated with combinatory chemotherapy (14%). No one received any further line of therapy (Figure 1C).

**Figure 2:**
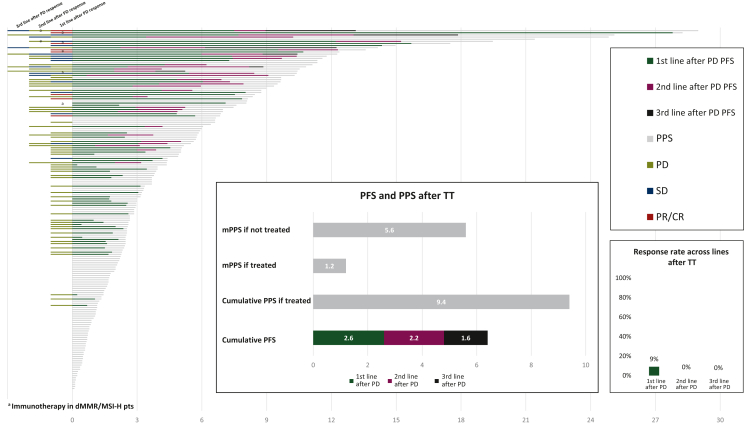
**Progression-free survival, postprogression survival, and overall response rate across treatments after progression to TT**. CR, complete response; dMMR, deficient mismatch repair; MSI-H, microsatellite instable-high; PD, progressive disease; PFS, progression-free survival; PPS, postprogression survival; PR, partial response; pts, patients; SD, stable disease; TT, targeted therapy.

The median follow-up from the time of PD to TT was 15 months (95% CI 10.3-28.3 months). The mPFS in the first, second, and third lines after TT were 2.6, 2.2, and 1.6 months, respectively, with objective responses observed in eight patients (9%) in the first line administered after progression to TT, including three patients with dMMR/MSI-H tumors responding to immunotherapy, and no responses in subsequent lines (Figure 2). The median PPS was 2.7 months (95% CI 2.4-3.2 months) in the whole population included in the analysis (Supplementary Figure S1A, available at https://doi.org/10.1016/j.esmoop.2024.102996) and favored those receiving at least one systemic line of treatment (*n* = 85) over those not eligible for further active treatments (*n* = 94) [5.6 versus 1.2 months, respectively (HR 0.18, 95% CI 0.12-0.26; *P* < 0.0001); Supplementary Figure S1B, available at https://doi.org/10.1016/j.esmoop.2024.102996].

**Figure 3:**
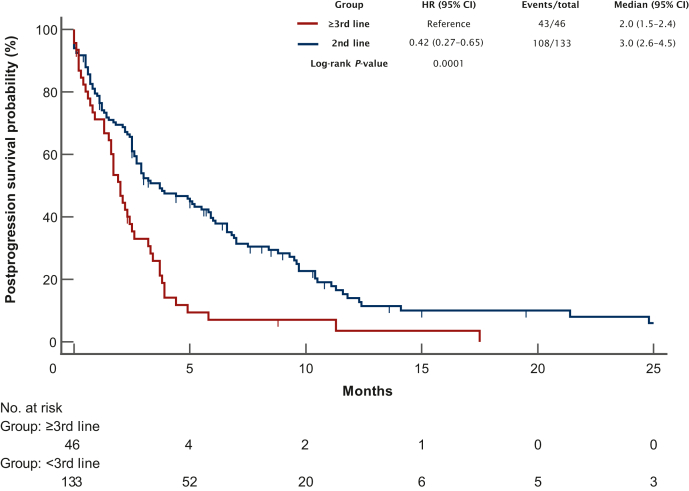
**Postprogression survival according to the line of administration of TT.** CI, confidence interval; HR, hazard ratio; TT, targeted therapy.

The potential prognostic factors associated with outcome were then investigated and PPS was found to be significantly longer in patients who had received TT in the second line as compared with those exposed to the same treatment in the third line or beyond (mPFS: 3.0 versus 2.0 months, HR 0.42, 95% CI 0.27-0.65; *P* = 0.0001; Figure 3). Notably, this correlation retained statistical significance in a multivariate Cox regression model (HR 0.53, 95% CI 0.34-0.83; *P* < 0.01), together with the presence of ascites at the time of PD to TT (HR 1.62, 95% CI 1.09-2.41; *P* = 0.02), after adjustment for other potential confounders. By contrast, metastatic spread to the peritoneum (*P* = 0.11), or to two or more organs (*P* = 0.07), and the documentation of radiological response during previous TT (*P* = 0.29) did not yield statistical significance in the multivariate model (Table 1).

**Table 1:** Univariate and multivariate analyses for postprogression survival

Characteristics	*n*	Postprogression survival				
		Median (months)	Univariate analysis		Multivariate analysis	
			HR (95% CI)	*P*	HR (95% CI)	*P*
**Sex**						
Male	73	2.5	1.19 (0.86-1.64)	0.31	—	—
Female	106	2.9			—	—
**Sidedness**					—	—
Right	119	2.6	0.97 (0.68-1.37)	0.85	—	—
Left/rectum	60	2.8			—	—
**Mucinous histology**					—	—
Yes	61	2.5	0.91 (0.64-1.28)	0.57	—	—
No	110	2.8			—	—
NA	8	—	—	—	—	—
**Synchronous**						
Yes	128	2.5	1.16 (0.81-1.66)	0.41	—	—
No	51	2.9			—	
**Line of TT**						
Second	133	3.0	0.49 (0.34-0.71)	**<0.001**	0.53 (0.34-0.83)	**<0.01**
Third and beyond	46	2.0				
**Peritoneal metastases at the time of progression to TT**						
Yes	109	2.7	1.78 (1.21-2.63)	**0.004**	1.47 (0.92-2.36)	0.11
No	51	3.0				
NA	19	—	—	—	—	—
**Number of organs involved at the time of progression to TT**						
≥3	103	2.5	1.93 (1.34-2.78)	**<0.001**	1.47 (0.97-2.23)	0.07
<3	57	4.4				
NA	19	—	—	—	—	—
**New organs involved at the time of progression to TT**						
Yes	57	2.8	1.04 (0.73-1.48)	0.83	—	—
No	100	2.9			—	
NA	22	—	—	—	—	—
**Ascites at the time of progression to TT**						
Yes	54	2.4	1.94 (1.35-2.79)	**<0.001**	1.62 (1.09-2.41)	**0.02**
No	99	3.7				
NA	26	—	—	—	—	—
**MEK inhibitor administered**						
Yes	38	2.4	1.22 (0.83-1.79)	0.31	—	—
No	141	2.9			—	
**Previous response to TT**						
PR/CR	34	3.5	0.61 (0.39-0.95)	**0.03**	0.77 (0.48-1.25)	0.29
SD/PD	144	2.5				
NA	1	—	—	—	—	—

Bold: *P*-value < 0.05.

CI, confidence interval; CR, complete response; HR, hazard ratio; NA, not available; PD, progression disease; PR, partial response; SD, stable disease; TT, targeted therapy.

**Figure 4:**
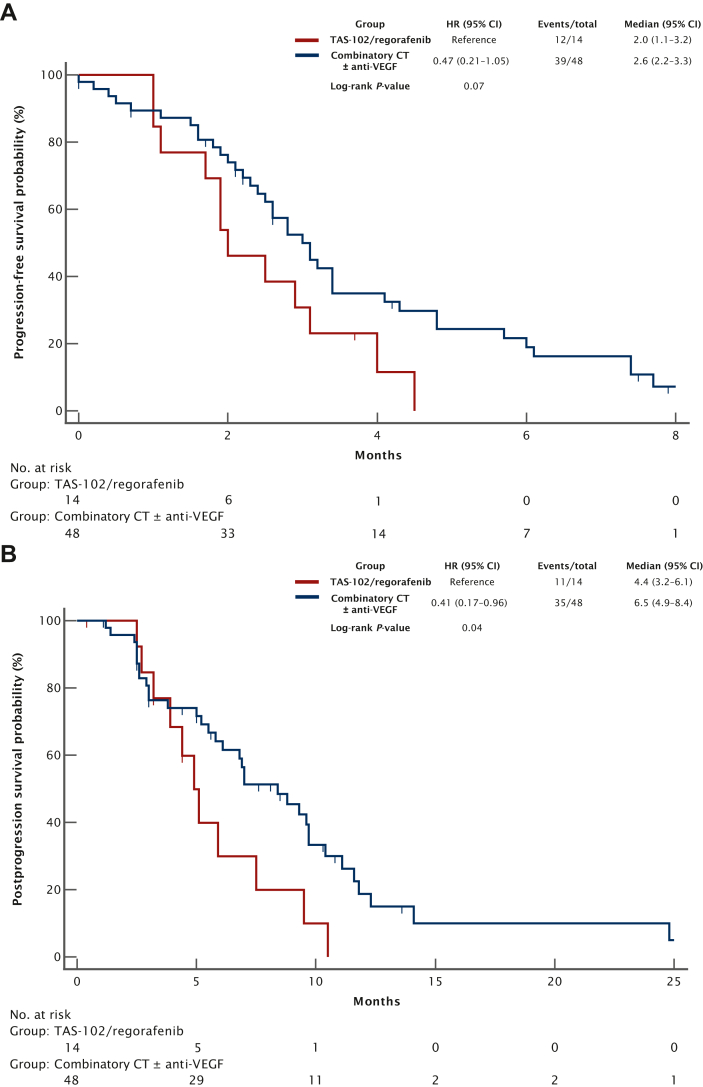
**(A) Progression-free survival and (B) postprogression survival with combinatory chemotherapy ± anti-VEGF versus regorafenib or trifluridine–tipiracil in the first line after progression to TT in patients with pMMR/MSS.** CI, confidence interval; CT, chemotherapy; HR, hazard ratio; pMMR, proficient mismatch repair; MSS, microsatellite stable; TAS-102, trifluridine–tipiracil; TT, targeted therapy; VEGF, vascular endothelial growth factor.

Forty-eight (77%) out of 62 patients with proficient mismatch repair (pMMR)/microsatellite stable (MSS) treated with TT in the second-line received combinatory chemotherapy ± anti-VEGF and 14 (23%) trifluridine–tipiracil (*n* = 10) or regorafenib (*n* = 4). Overall, chemotherapy ± anti-VEGF resulted in longer PFS and PPS compared with trifluridine–tipiracil or regorafenib, respectively (mPFS: 2.6 versus 2.0 months; HR 0.47, 95% CI 0.21-1.05; *P* = 0.07; Figure 4A; median PPS: 6.5 versus 4.4 months, HR 0.41, 95% CI 0.17-0.96; *P* = 0.04; Figure 4B). The addition of an antiangiogenic agent (*n* = 37) was associated with longer PFS and PPS (*P*_PFS_ = 0.07 and *P*_PPS_ = 0.09; Supplementary Figure S2A and B, available at https://doi.org/10.1016/j.esmoop.2024.102996).

Two patients were exposed to TT after the evidence of PD. Among them, one experienced oligoprogression in the liver and received radiofrequency ablation while continuing BRAF and EGFR blockade, achieving a clinically meaningful PFS of 14.3 months. The other received TT after a treatment break of 1 month but did not achieve any benefit and died shortly after TT reintroduction (PPS: 1.7 months).

## Discussion

The introduction of the targeted approach in the treatment of *BRAF*^*V600E*^-mutated mCRC led to a clinically meaningful OS improvement. However the duration of disease control is rather short (mPFS: 4.3 months), and only limited evidence is available to drive the choice of subsequent systemic therapies when feasible.^6^^,^^9^^,^^10^

Actually, we found that less than half of patients receiving TT are eligible for at least one subsequent line of treatment, which is consistent with previous reports,^14^^,^^15^ with a median PPS of ∼5 months, as compared with ∼1 month in those not receiving further therapies, thus emphasizing the limited benefit from postprogression treatments. Among them, combinatory chemotherapy was the preferred option (59%), with trifluridine–tipiracil and regorafenib being the preferred strategy in subsequent lines (50% and 86% in the second and third lines after TT). In the *post hoc* analysis of the BEACON CRC trial, trifluridine–tipiracil and regorafenib were largely underrepresented (12% and 28% after progression to TT in second- and third-line, respectively), probably due to the timeframe of study enrollment.^14^

It is noteworthy that earlier administration of TT in the *continuum-of-care* of patients with *BRAF*^*V600E*^-mutated mCRC was an independent predictor of longer PPS. This is likely due to the higher percentage of patients treated with TT in the second line being able to receive at least one line of therapy after PD (47%), as compared with those receiving TT in the third or later lines (30%). Furthermore, patients treated with TT earlier in their *continuum-of-care* were more likely to receive a combinatory cytotoxic irinotecan- or oxaliplatin-based strategy after PD (69%), instead of trifluridine–tipiracil or regorafenib. These findings confirm previous data from molecularly unselected patients with mCRC.^16^

We also reported an additional benefit from VEGF blockade in terms of PFS and PPS when associated with cytotoxic regimens compared with other treatment strategies in patients with pMMR/MSS, thus confirming the efficacy of antiangiogenic strategies in this subgroup. The continuation of TT beyond radiological progression was infrequent (*n* = 2), with a signal of prolonged disease control in one case of oligoprogression and no benefit after a brief TT-free interval in the other case, suggesting that accurate selection of patients is needed to tailor the continuation/reintroduction strategy after previous failure, possibly including molecular analyses, in accordance with growing evidence accumulated in the last years.^17^, ^18^, ^19^

In fact, *post hoc* translational analyses of longitudinal blood samples collected at baseline and PD in patients enrolled in the BEACON and EVIC trials showed that roughly two-thirds of *BRAF*^*V600E*^*-*mutated tumors develop acquired alterations in the mitogen-activated protein kinase (MAPK) pathway as a mechanism of resistance to combined BRAF and EGFR blockade,^20^ and some of them may even benefit from other targeted strategies.^21^^,^^22^ Overall, these preliminary data suggest that liquid biopsy may serve as a noninvasive screening tool for the reuse of TT in patients with *BRAF*^*V600E*^*-*mutated mCRC. This approach, which proved promising in *RAS* and *BRAF* wild-type patients candidate for anti-EGFR re-treatment,^23^^,^^24^ showed encouraging signals of activity also in some case series of patients with *BRAF*^*V600E*^*-*mutated mCRC reexposed to TT after excluding potential drivers of resistance in their circulating tumor DNA,^25^^,^^26^ and may be an appealing approach for future studies.

Clear limitations of our work are its retrospective nature and the lack of a risk–benefit assessment, as safety information related to treatments after PD to TT was not collected. Furthermore, our work suffers from a high risk of immortality bias because those patients with the most aggressive disease had likely passed away before receiving TT in the second or later lines. We also acknowledge that the percentage of patients with dMMR/MSI-H tumors in our cohort was small (11%), possibly due to the low rate of occurrence of PD events among patients receiving first-line immunotherapy, thus limiting the need to receive subsequent TT in the timeframe of our analysis. Notably, only the 68% of patients with dMMR/MSI-H tumors in our cohort were exposed to ICIs, and even fewer (26%) before TT, with only one case as a first-line regimen, in contrast to current international guidelines.^13^^,^^27^ This is likely due to the timespan of data collection that started almost 3 years before the availability of pembrolizumab in Italy as the first-line option in dMMR/MSI-H mCRC. Moreover, no patient in our series received trifluridine–tipiracil plus bevacizumab after TT, which more recently emerged as a new standard regimen in the third-line treatment of mCRC.^13^^,^^27^ In addition, if the ongoing randomized phase III BREAKWATER trial, investigating first-line TT with or without chemotherapy versus standard chemotherapy in pMMR/MSS and patients with *BRAF*^*V600E*^*-*mutated mCRC, demonstrates higher efficacy of combinatory TT plus chemotherapy, the use of the TT will be anticipated in first-line and present data might be less clinically relevant.^28^

Despite these limitations, this is the largest series of patients with *BRAF*^*V600E*^-mutated mCRC with detailed follow-up data collected after progression to previous TT, and clinically relevant messages can be drawn from this work. First, our data further support the recommendation of international guidelines to consider TT administration immediately after progression to the first line; second, from a clinical perspective, we provide support to physicians in the therapeutic decision-making after PD to TT in pMMR/MSS tumors, highlighting that combinatory regimens plus anti-VEGF are the preferred strategy when feasible; third, the description of real-world attrition rates and efficacy data after TT in patients with *BRAF*^*V600E*^-mutated mCRC may be helpful in the design of clinical trials focused on the *continuum-of-care* of this specific molecular subgroup of patients, including rechallenge strategies.^29^
