# Supplementary figures and images for: Treatment of patients with BRAFV600E-mutated metastatic colorectal cancer after progression to encorafenib and cetuximab: data from a real-world nationwide dataset
Source: ESMO Open. 2024 Apr 12;9(4):102996. doi: 10.1016/j.esmoop.2024.102996 (PMC11024565; doi:10.1016/j.esmoop.2024.102996)

A

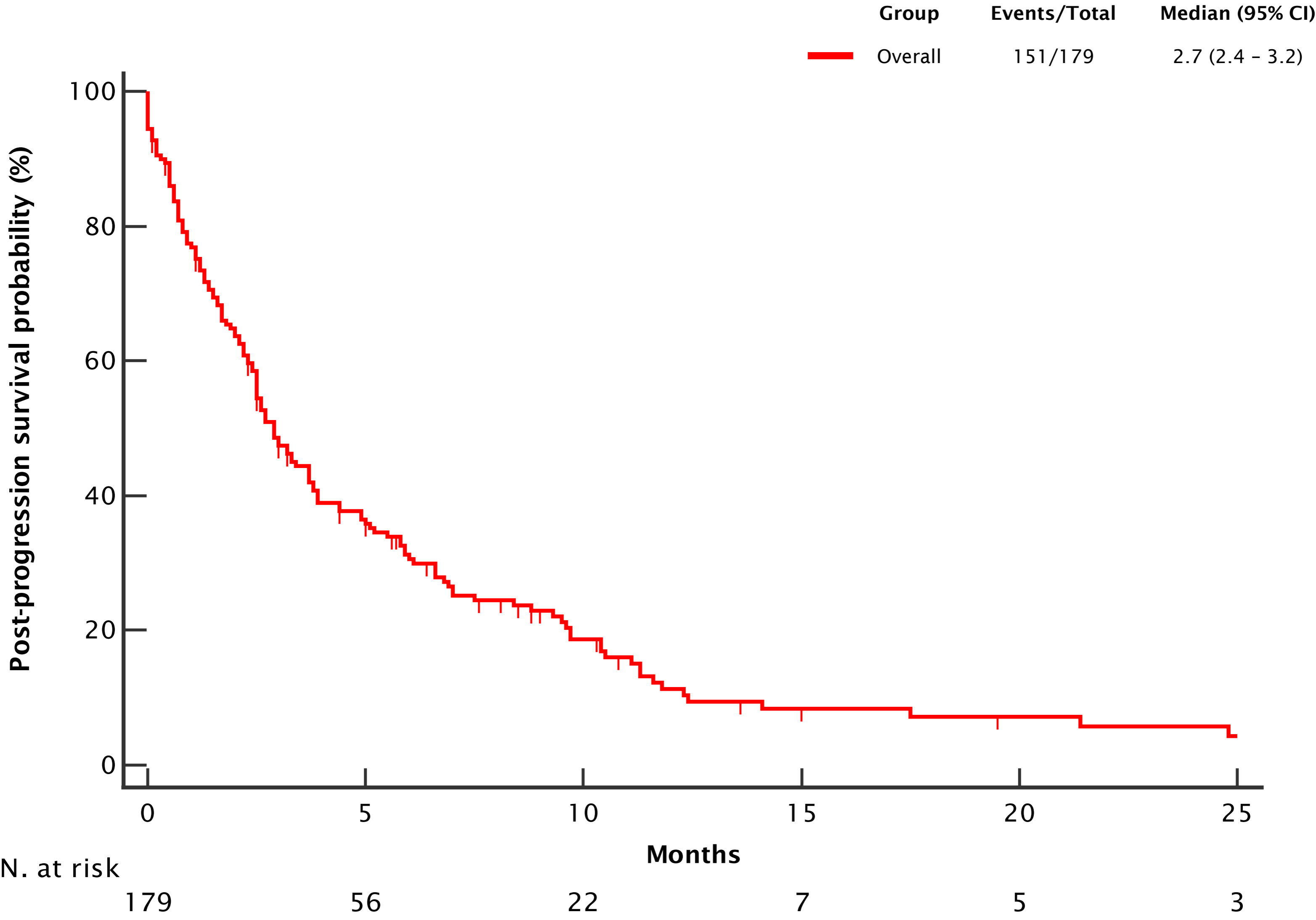

B

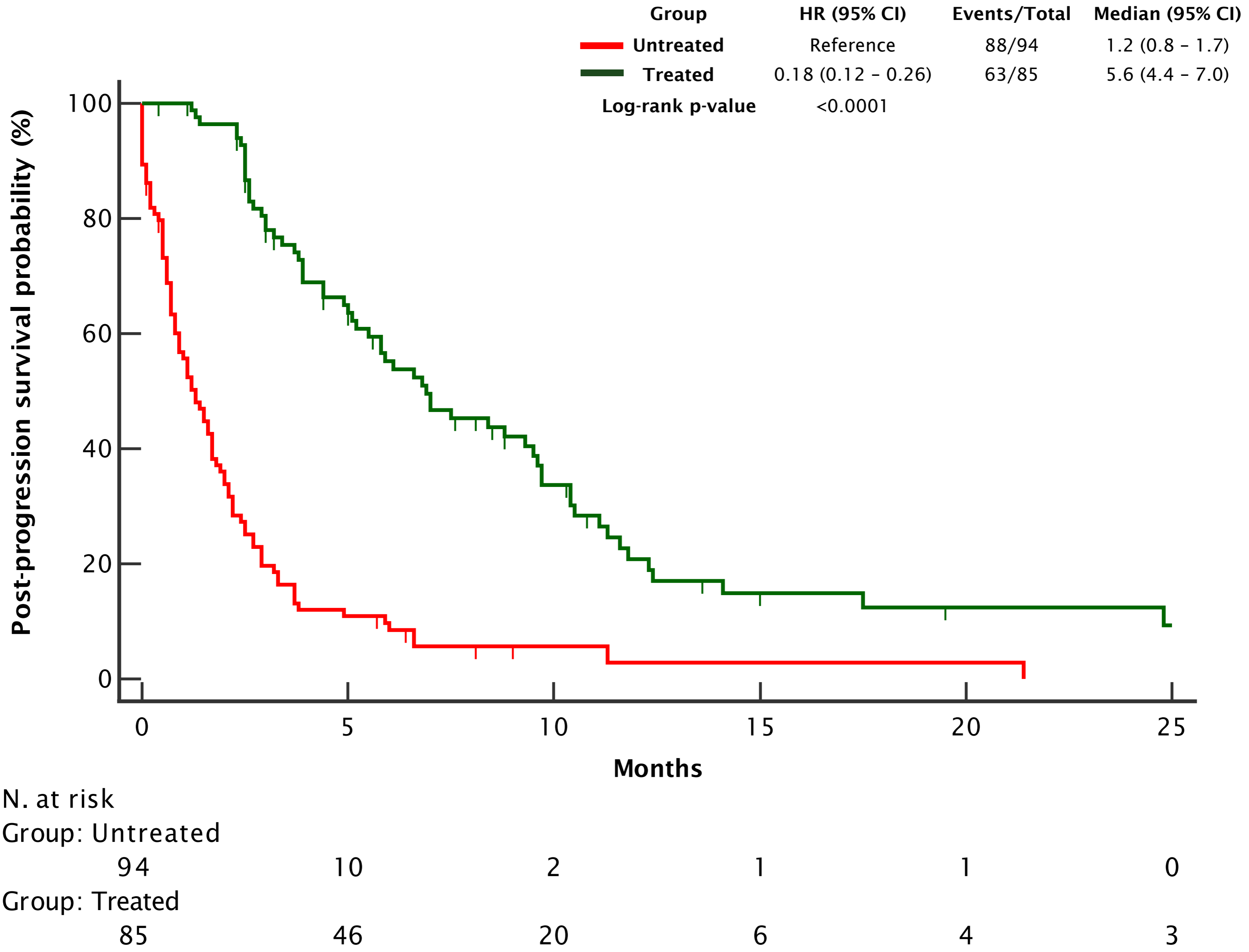

Supplement: Supplementary Figure 1 [file mmc1.pdf]

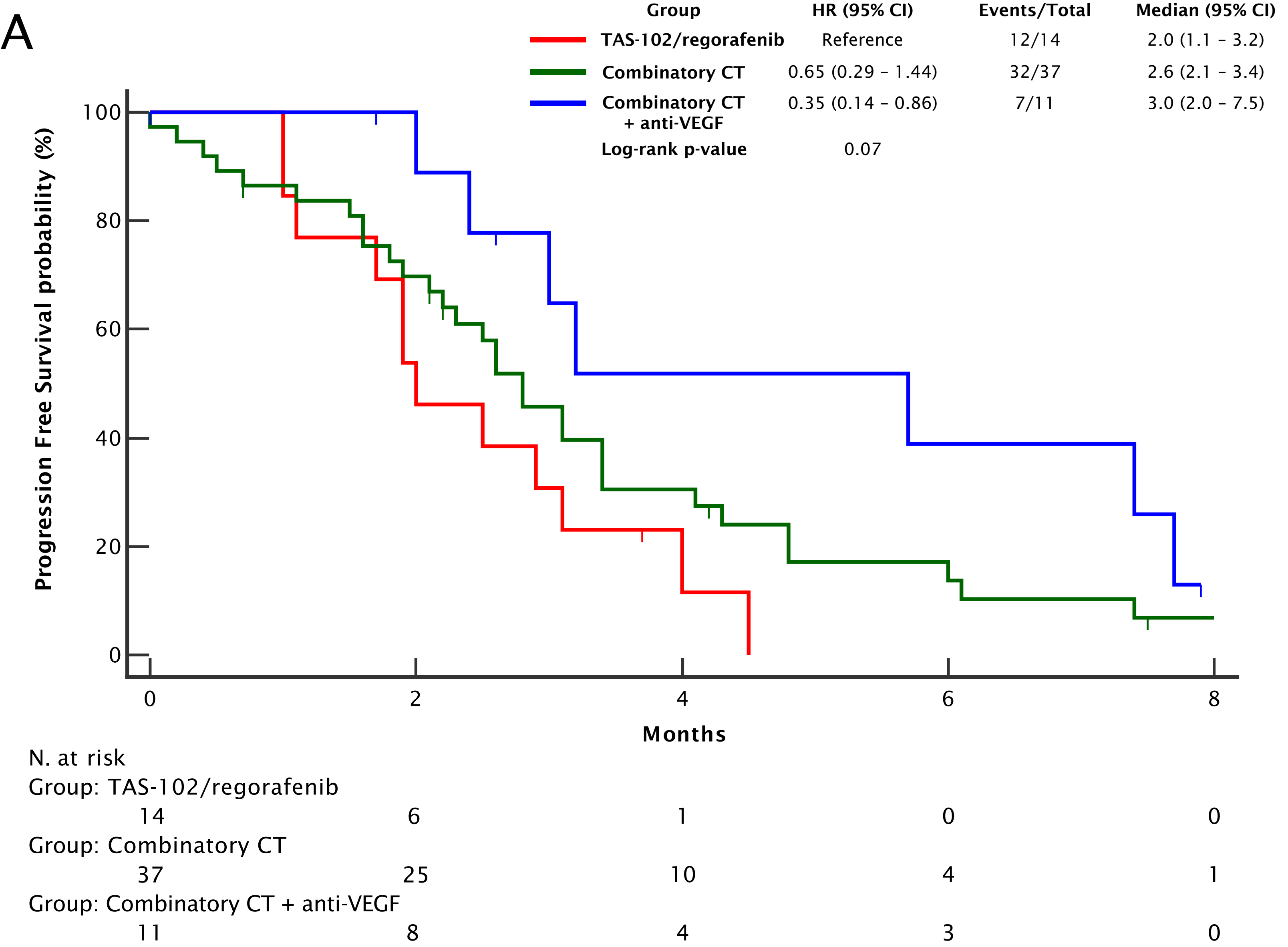

Supplement: Supplementary Figure 2 [file mmc2.pdf]
