# Supplementary material for: Treatment of patients with BRAFV600E-mutated metastatic colorectal cancer after progression to encorafenib and cetuximab: data from a real-world nationwide dataset
Source: ESMO Open. 2024 Apr 12;9(4):102996. doi: 10.1016/j.esmoop.2024.102996 (PMC11024565; doi:10.1016/j.esmoop.2024.102996)
Supplement: Supplementary Figure Legend [file mmc3.docx]

**Supplementary Figure legend**

**Supplementary Figure 1. PPS in the overall population (A) and according to any treatment received after progression *versus* none (B).**

CI: confidence interval; HR: hazard ratio

**Supplementary Figure 2. Progression-free survival (A) and post-progression survival (B) with combinatory chemotherapy + anti-VEGF *versus* combinatory chemotherapy *versus* regorafenib or trifluridine-tipiracil in the first line after progression to TT in pMMR/MSS patients.**

CI: confidence interval; CT: chemotherapy; HR: hazard ratio, N: number; TAS-102: trifluridine-tipiracil, VEGF: Vascular Endothelial Growth Factor
