# Supplementary material for: Treatment of patients with BRAFV600E-mutated metastatic colorectal cancer after progression to encorafenib and cetuximab: data from a real-world nationwide dataset
Source: ESMO Open. 2024 Apr 12;9(4):102996. doi: 10.1016/j.esmoop.2024.102996 (PMC11024565; doi:10.1016/j.esmoop.2024.102996)
Supplement: Supplementary Table 1 [file mmc4.docx]

| **Supplementary table 1: baseline characteristics** | | |
| --- | --- | --- |
| **Characteristics** | **Total (n=179)** | **n. (%)** |
| **Age at the diagnosis of CRC** | Median age (range) | 64 (25-84) |
| **Sex** | Male  Female | 73 (41)  106 (59) |
| **Primary tumor location** | Left colon and rectum  Right colon | 60 (34)  119 (66) |
| **Diagnosis of metastatic disease synchronous to primary tumour (< 6 months)** | Yes  No | 128 (71)  51 (29) |
| **Primary tumour resected at diagnosis** | Yes  No | 126 (70)  53 (30) |
| **MSI/MMR status** | High/dMMR  Low/pMMR  NA | 19 (11)  156 (89)  4 |
| **Mucinous histology** | Yes  No  NA | 61 (36)  110 (64)  8 |
| **Previous treatment regimens** | Oxaliplatin-based  Irinotecan-based  Anti-VEGF  Anti-EGFRs  Immunotherapy  Trifluridine-tipiracil  Regorafenib | 165 (92)  107 (60)  136 (76)  12 (7)  5 (3)  9 (5)  5 (3) |
| **First-line regimen** | Triplet regimen  Doublets  Monochemotherapy  Immunotherapy | 57 (32)  109 (61)  12 (7)  1 (<1) |
| **Line of administration of encorafenib and cetuximab** | Second  Third and beyond | 133 (75)  46 (25) |
| **Previous MEK inhibitor administered** | Yes  No | 38 (21)  141 (79) |
| **RECIST response to encorafenib and cetuximab** | CR  PR  SD  PD  NA | 2 (1)  32 (18)  80 (45)  64 (36)  1 |
| **PFS to encorafenib and cetuximab** | Median months (95% CI) | 4.7 (4.1 – 5.2) |
| **Peritoneal metastases after encorafenib and cetuximab** | Yes  No  NA | 109 (68)  51 (32)  19 |
| **Ascites after encorafenib and cetuximab** | Yes  No  NA | 54 (35)  99 (65)  26 |
| **Number of organs involved after encorafenib and cetuximab** | ≥3  <3  NA | 103 (64)  57 (36)  19 |
| **New organs involved after encorafenib and cetuximab** | Yes  No  NA | 57 (36)  100 (64)  22 (12) |
| **CEA** | ≥10  <10  NA | 72 (65)  39 (35)  68 |
| **Ca 19.9** | ≥40  <40  NA | 68 (65)  37 (35)  74 |

**Legend**

CEA: carcinoembryonic antigen; CI: confidence interval; CR: complete response; CRC: colorectal cancer; dMMR: deficient mismatch repair; EGFR: Epithelial Growth Factor Receptor; MMR: mismatch repair; MSI: microsatellite; N: number; NA: not available; PD: progressive disease; PFS: progression-free survival; pMMR: proficient mismatch repair; PR: partial response; RECIST: Response evaluation criteria in solid tumors; SD: stable disease; VEGF: Vascular Endothelial Growth Factor
